# Unveiling the origins of elastic anisotropy and thermodynamic stability in Mg Zn alloy strengthening phases via first principles

**DOI:** 10.1038/s41598-025-96708-x

**Published:** 2025-04-07

**Authors:** Zhiyong You, Shuaishuai Jin, Peide Han, Aoxue Jiang, Chunle Sun

**Affiliations:** Shanxi Key laboratory of Magnesium matrix materials, College of Materials Science and Engineering, Taiyuan University of Technology, Taiyuan, 030024 People’s Republic of China

**Keywords:** Elastic anisotropy, Thermal conductivity, Thermal expansion, Grüneisen parameter, Materials science, Theory and computation, Electronic structure

## Abstract

This study systematically investigates the elastic anisotropy and thermodynamic properties of $\:{{\upbeta\:}}_{1}^{{\prime\:}}$ phase in Mg-Zn alloys through first-principles calculations combined with Debye-Grüneisen theory. Three critical intermetallic phases - monoclinic Mg_4_Zn_7_, cubic MgZn_2_ (C-MgZn_2_), and hexagonal MgZn_2_ (H-MgZn_2_) phases were comparatively analyzed. Electronic structure analysis reveals that C-MgZn_2_ and H-MgZn_2_ exhibit stronger chemical bonding stability compared to Mg_4_Zn_7_. Phonon dispersion characteristics demonstrate distinct vibrational patterns: C-MgZn_2_ and Mg_4_Zn_7_ display enhanced phonon modes at both low and high frequency ranges, while H-MgZn_2_ shows predominant medium-frequency vibrational modes. Elastic anisotropy evaluation identifies Mg_4_Zn_7_ as moderately anisotropic, H-MgZn_2_ as significantly anisotropic, and C-MgZn_2_ as nearly isotropic. Thermodynamic analysis predicts superior thermal stability for C-MgZn_2_, evidenced by its highest Debye temperature (θ_d_ = 366 K), maximum sound velocity (v_m_=3.468 m/s), and minimal Grüneisen parameter (γ = 0.641), correlating with its exceptional thermal conductivity. In contrast, Mg_4_Zn_7_ exhibits the highest thermal expansion coefficient among the investigated phases. These findings establish fundamental structure-property relationships that advance the understanding of $\:{{\upbeta\:}}_{1}^{{\prime\:}}$ phase stabilization mechanisms, providing critical guidance for designing high-performance Mg-Zn alloys through phase engineering strategies.

## Introduction

Magnesium alloys are recognized as the lightest structural metallic materials, and they have been extensively researched due to their potential applications in the aerospace and automotive industries, as weight reduction is critical for lowering energy costs and carbon dioxide emissions^1–4^. However, the strength and performance of alloys largely depend on the precipitate phases in their microstructures, particularly the metastable G.P. zones (Guinier-Preston zones) as well as $\:{{\upbeta\:}}_{1}^{{\prime\:}}$ phases and $\:{{\upbeta\:}}_{2}^{{\prime\:}}$ precipitates. The formation, structure, and interaction of these metastable phases with the matrix directly affect the strengthening mechanisms and performance of the alloys. Nonetheless, there are still controversies regarding the structures and evolution mechanisms of these precipitates^5,6^.

The $\:{{\upbeta\:}}_{1}^{{\prime\:}}$ precipitates are rod-shaped, grow parallel to the [0001]_α−Mg_ direction, and form at high temperatures^7^. They significantly enhance the strength of magnesium alloys, but their complex crystal structures have not been fully defined. Gao and Nie^8^discovered through electron microscopy that $\:{{\upbeta\:}}_{1}^{{\prime\:}}\:$rod-shaped precipitates actually have a monoclinic crystal structure similar to Mg_4_Zn_7_, rather than the traditional C14 structure. Their study also reported the orientation relationship between this structure and the magnesium matrix, which was confirmed by Singh and Tsai^9^. Rosalie^10^ specifically studied the orientation relationships of the C14 Laves phase MgZn_2_ and the Mg_4_Zn_7_ phase at grain boundaries and within grains. These two phases contain icosahedral quasicrystalline I phase atomic clusters in their structures. Within the twin boundaries, the orientation of the C14 Laves phase MgZn_2_ relative to the matrix is (11–20)_α−Mg_||(01–10)_C14_.

Hongbo Xie^11^ finally determined the internal structure of $\:{{\upbeta\:}}_{1}^{{\prime\:}}$ phase : it consists of two distinct two-dimensional unit cells (a rhombus structure with a 72-degree angle and an equilateral hexagonal structure with a 72-degree angle), which are arranged periodically along the direction of the fivefold symmetry axis. These 2D structures self-assemble into nanodomains containing 2D fivefold symmetry, C14, and C15 structures. The phase is neither a traditional crystal nor a known quasicrystal.

Bendo et al.^12^ confirmed through HAADF-STEM that in addition to C14 MgZn_2_ and monoclinic Mg_4_Zn_7_, $\:{{\upbeta\:}}_{1}^{{\prime\:}}$ precipitates exhibit various crystal structures. These structures consist of elongated hexagonal units of Mg_6_Zn_7_ and rhombic units of MgZn_2_, forming complex two-dimensional arrangements that include C14 and C15 Laves phases, quasi-crystals, and their approximants. Wang^13^, Liu^14^, Cheng^15^, and Du Cheng^16^ studied the structure of this $\:{{\upbeta\:}}_{1}^{{\prime\:}}$ phase using first-principles calculations combined with the cluster expansion (CE) method, finding that the $\:{{\upbeta\:}}_{1\:}^{{\prime\:}}\:$phase consists of monoclinic Mg_4_Zn_7_, cubic MgZn_2_ (C-MgZn_2_), and hexagonal MgZn_2_ (H-MgZn_2_), and pointed out that over-aging transforms these phases fully into (H-MgZn_2_).

Yi Yang^17^ conducted an in-depth analysis of the precipitation kinetics of magnesium-1.7at.% zinc alloy aged at 150 °C and 200 °C by comprehensively using characterization techniques such as thermoelectric power (TEP), X-ray diffraction, atom probe tomography (APT), and transmission electron microscopy. The study revealed that during the early aging stage, the $\:{\:{\upbeta\:}}_{1}^{{\prime\:}}$phase (rod-like precipitates) predominates, while in the over-aged stage, the $\:{{\upbeta\:}}_{2}^{{\prime\:}}$ phase (plate-like precipitates) takes the lead. Based on classical nucleation and growth theory, the research developed models suitable for rod-like and plate-like precipitates, detailing the competitive relationship between the two types of precipitates. Finally, a time-temperature-transformation (TTT) diagram for this alloy system was proposed, providing new insights and theoretical support for understanding the precipitation behavior in magnesium-zinc alloys.

The development of high-performance magnesium-zinc alloys necessitates fundamental understanding of$\:{\:\:{\upbeta\:}}_{1}^{{\prime\:}}$ phase evolution mechanisms. Although extensive studies have characterized rod-shaped $\:{\:{\upbeta\:}}_{1}^{{\prime\:}}$(Mg_4_Zn_7_) and plate-like $\:{\:{\upbeta\:}}_{2}^{{\prime\:}}$ precipitates in Mg-Zn systems, critical knowledge gaps persist. To address these challenges, we employ density functional theory (DFT) coupled with Debye-Grüneisen theory for systematic evaluation of three dominant $\:{\:{\upbeta\:}}_{1}^{{\prime\:}}$phase variants: monoclinic Mg_4_Zn_7_, cubic MgZn_2_(C-MgZn_2_), and hexagonal MgZn_2_ (H-MgZn_2_). This work specifically targets three unresolved issues: (1) quantifying the chemical bonding disparities governing phase stability, (2) decoding the correlation between elastic anisotropy and precipitate morphology evolution, and (3) establishing thermodynamic fingerprints (Debye temperature, Grüneisen parameter) for phase selection predictions. Our computational strategy integrates electronic structure analysis, phonon dispersion calculations, and anisotropic elasticity modeling, providing multiscale insights to reconcile existing experimental controversies .

## Theoretical methods and computational details

Zener first proposed the anisotropy coefficient A factor for cubic lattices^18^:

1 $$A=\frac{{2{C_{44}}}}{{{C_{11}} - {C_{12}}}}$$

The anisotropic constants A_B_ and A_G_, which can be evaluated based on the bulk modulus and shear modulus, were introduced by Chung and Buessem^19^ in 1967, refining Zener’s model. However, these three anisotropy factors neglect the influence of elastic stiffness and are only applicable to cubic crystal systems.

2 $${A_B}=\frac{{{B_V} - {B_R}}}{{{B_V}+{B_R}}}$$

3 $${A_G}=\frac{{{G_V} - {G_R}}}{{{G_V}+{G_R}}}$$

Shivakumar^20^ introduced a comprehensive anisotropy index AU, which can be used to analyze anisotropy coefficients in hexagonal, trigonal, monoclinic, and other crystal systems. When AU = 0, the material is isotropic; when AU > 0, the material is anisotropic.

4 $${A^U}=5\frac{{{G_V}}}{{{G_R}}}+\frac{{{B_V}}}{{{B_R}}} - 6 \geqslant 0$$

The shear anisotropy factors^21^ A_1_, A_2_, and A_3_ are calculated using the following formulas, representing the shear anisotropy factors on the (100), (010), and (001) planes, respectively. The crystal is isotropic only when A_1_ = A_2_ = A_3_=1:

5 $${A_1}=\frac{{4{C_{44}}}}{{{C_{11}}+{C_{33}} - 2{C_{13}}}}$$

6 $${A_2}=\frac{{4{C_{55}}}}{{{C_{22}}+{C_{33}} - 2{C_{23}}}}$$

7 $${A_3}=\frac{{4{C_{66}}}}{{{C_{11}}+{C_{22}} - 2{C_{12}}}}$$

Based on the first-principles and the Debye–Grüneisen model theory^22^, the free energy G can be calculated by ignoring electronic entropy:

8 $${\text{G(T)=}}{{\text{H}}_{\text{0}}}{\text{+}}{{\text{E}}_{\text{D}}}{\text{(T)-T}}{{\text{S}}_{\text{D}}}{\text{(T)}}$$

9 $${E_D}(T)=3{k_{\text{B}}}TD\left( {\frac{{{\theta _{\text{d}}}}}{T}} \right)+\frac{9}{8}{k_{\text{B}}}{\theta _{\text{d}}}$$

10 $${S_{\text{D}}}(T)=3{k_{\text{B}}}\left\{ {\frac{4}{3}D\left( {\frac{{{\theta _{\text{d}}}}}{T}} \right) - \ln \left[ {1 - \exp \left( { - \frac{{{\theta _{\text{d}}}}}{T}} \right)} \right]} \right\}$$

11 $$D=\frac{n}{{{x^n}}}\int_{0}^{x} {\frac{{{t^3}}}{{{{\text{e}}^t} - 1}}} ~{\text{d}}t$$

12 $$G(T)={\text{ }}{H_0}+3{k_{\text{B}}}T\ln \left[ {1 - \exp \left( { - \frac{{{\theta _{\text{d}}}}}{T}} \right)} \right]+\frac{9}{8}{k_{\text{B}}}{\theta _{\text{d}}} - {k_{\text{B}}}TD\left( {\frac{{{\theta _{\text{d}}}}}{T}} \right)$$

The non-harmonic effects of lattice vibrations can be expressed through the Grüneisen parameter:

13 $$\gamma =\frac{3}{2}\left( {\frac{{3v_{l}^{2} - 4v_{t}^{2}}}{{v_{l}^{2}+2v_{t}^{2}}}} \right)$$

The Debye temperature θ_d_ can be obtained, and the Grüneisen parameter can be used to calculate the isochoric heat capacity, isobaric heat capacity, and thermal expansion coefficient through the Debye–Grüneisen model:

14 $${C_V}=3{\kappa _{\text{B}}}\left[ {4D\left( {{\theta _{\text{d}}}/T} \right) - \frac{{3{\theta _{\text{d}}}/T}}{{\exp \left( {{\theta _{\text{d}}}/T} \right) - 1}}} \right]$$

15 $${C_P}={C_V}(1+\alpha \gamma T)$$

16 $$\alpha =\frac{{\gamma {C_V}}}{{{B_0}V}}$$

The thermal conductivity k_min_ is a characteristic of the material, indicating its ability to conduct heat. Cahill and Pohl^23^ suggest that the minimum thermal conductivity can be calculated as follows:

17 $${k_{\hbox{min} }}=\frac{{{k_B}}}{{2.48}}{n^{\frac{2}{3}}}\left( {2{v_t}+{v_l}} \right)$$

This study is based on the Vienna Ab-initio Simulation Package VASP v.6.3.2(https://www.vasp.at/)^24^ within the framework of density functional theory (DFT), employing the projector augmented wave (PAW) pseudopotential method^25^ and the Perdew-Burke-Ernzerhof (PBE) functional in the generalized gradient approximation (GGA)^26^ to describe the exchange-correlation interactions while accounting for electron spin effects. The cutoff energy for the plane wave basis set is set to 400 eV, and Brillouin zone integration is carried out using Gamma point sampling with a 4 × 4 × 1 k-point grid. During structural optimization, the atomic coordinates and cell volumes of all unit cells are relaxed using the Broyden-Fletcher-Goldfarb-Shanno (BFGS)^27^ self-consistent algorithm until the forces on all atoms decrease to below 0.015 eV/Å. For the elements Mg and Zn, the outermost electron configurations are Mg: s²p⁰ and Zn: d¹⁰p², respectively.

Phonopy v.2.37.0(https://phonopy.github.io/phonopy/)^28^ is used to perform phonon calculations on three phases using the finite displacement method.Utilizing linear response theory, the stress-strain method with an optimized high-efficiency strain matrix set (OHESS)^29^ was employed to compute the elastic constants and mechanical properties of the MgZn_2_ phase under linear strain, using Liu’s ELASTOOL v.3.62(https://github.com/gmp007/elastool) software package^30^. Visualization was performed using the VESTA v.3.5.8(https://jp-minerals.org/vesta/en/download.html) software package^31^.The thermodynamic properties of the phases are fitted using the Debye-Grüneisen theory in combination with phonon velocities and Debye temperatures calculated from the elastic properties.

## Results and discussions

### Stability of the $\:{{\upbeta\:}}_{1}^{{\prime\:}}$-phase

**Fig. 1:**
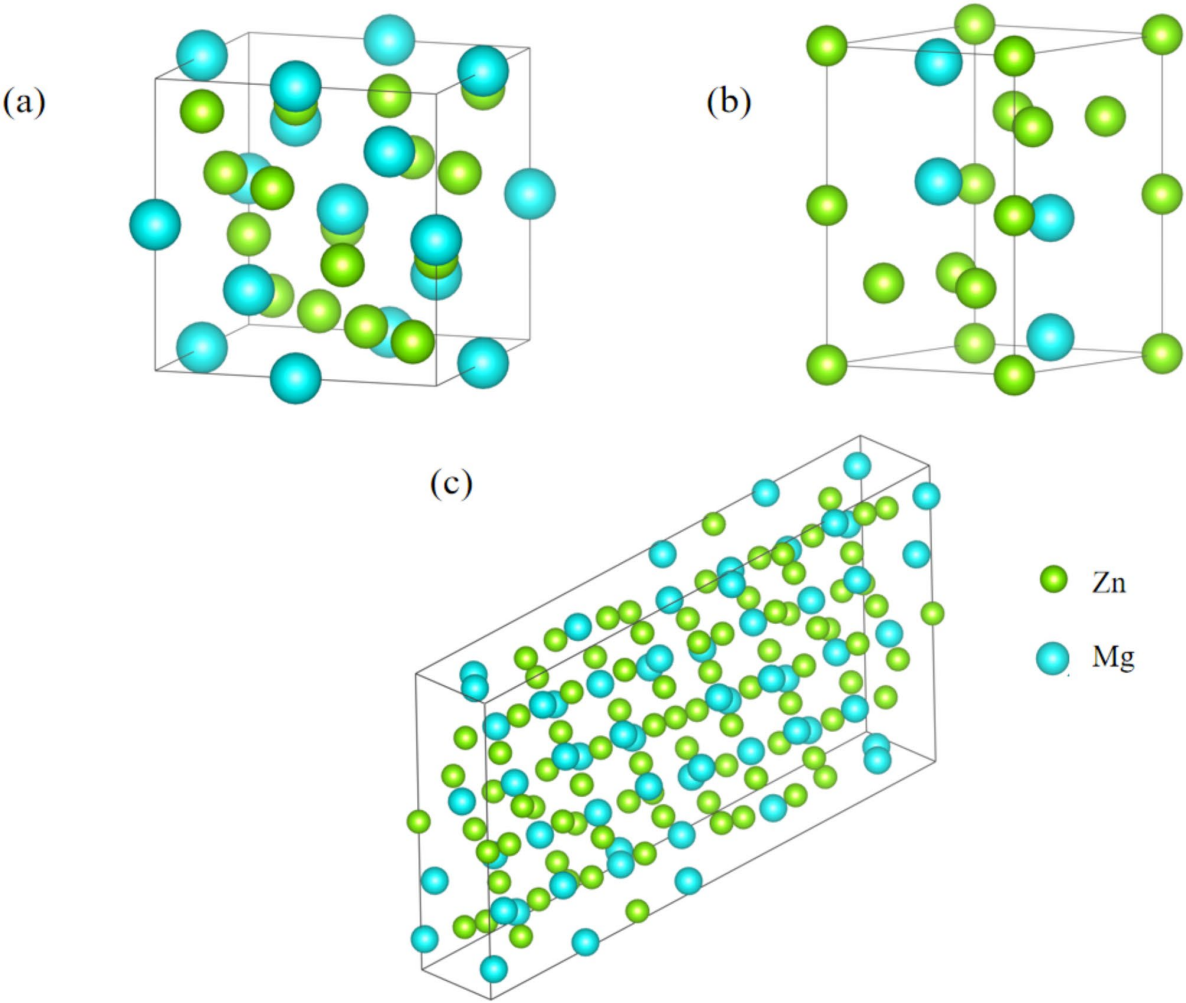
Crystal structures of C-MgZn_2_, H-MgZn_2_, Mg_4_Zn_7_.

According to the previous research^10–13^, the phase is a mixed structure of cubic C15 Laves phase MgZn_2_, hexagonal C14 Laves phase MgZn_2_, and monoclinic Mg_4_Zn_7_ phase.The structural model is shown in Fig. 1. As shown in Table 1, the formation energy difference among the three phases is 0.3 KJ/mol, with the lowest formation energy phase being H-MgZn_2_ and the highest being Mg_4_Zn_7_ phase. The phase with C14 structure is H-MgZn_2_. From the perspective of formation energy, the phase transformation could possibly be from the Mg_4_Zn_7_ phase and C-MgZn_2_ phase to H-MgZn_2_ phase^16^.

**Table 1:** Optimized structures and formation energy cohesion energies of C-MgZn_2_, H-MgZn_2_, Mg_4_Zn_7_.

	lattice constant(Å)			formation energy(KJ/mol)	cohesive energy (KJ/mol)	Reference
	a	b	c			
C-MgZn_2_	7.341	7.341	7.341	-12.820	98.143	This work
H-MgZn_2_	5.223	5.223	8.566	-12.965	97.998	This work
	5.221	5.221	8.503	-13.41	-	Ref.^16^
	5.230	5.230	8.470	-11.47	134.22	Ref.^32^
	5.187	5.187	8.561	-13.186	132.617	Ref.^33^
	5.251	5.251	8.445	-7.9	-	Ref.^34^
Mg_4_Zn_7_	25.960	5.240	14.280	-12.618	118.040	This work
	26.304	5.287	14.141	-13.32	-	Ref.^34^
	26.484	5.190	14.185	-13.02	-	Ref.^16^

**Fig. 2:**
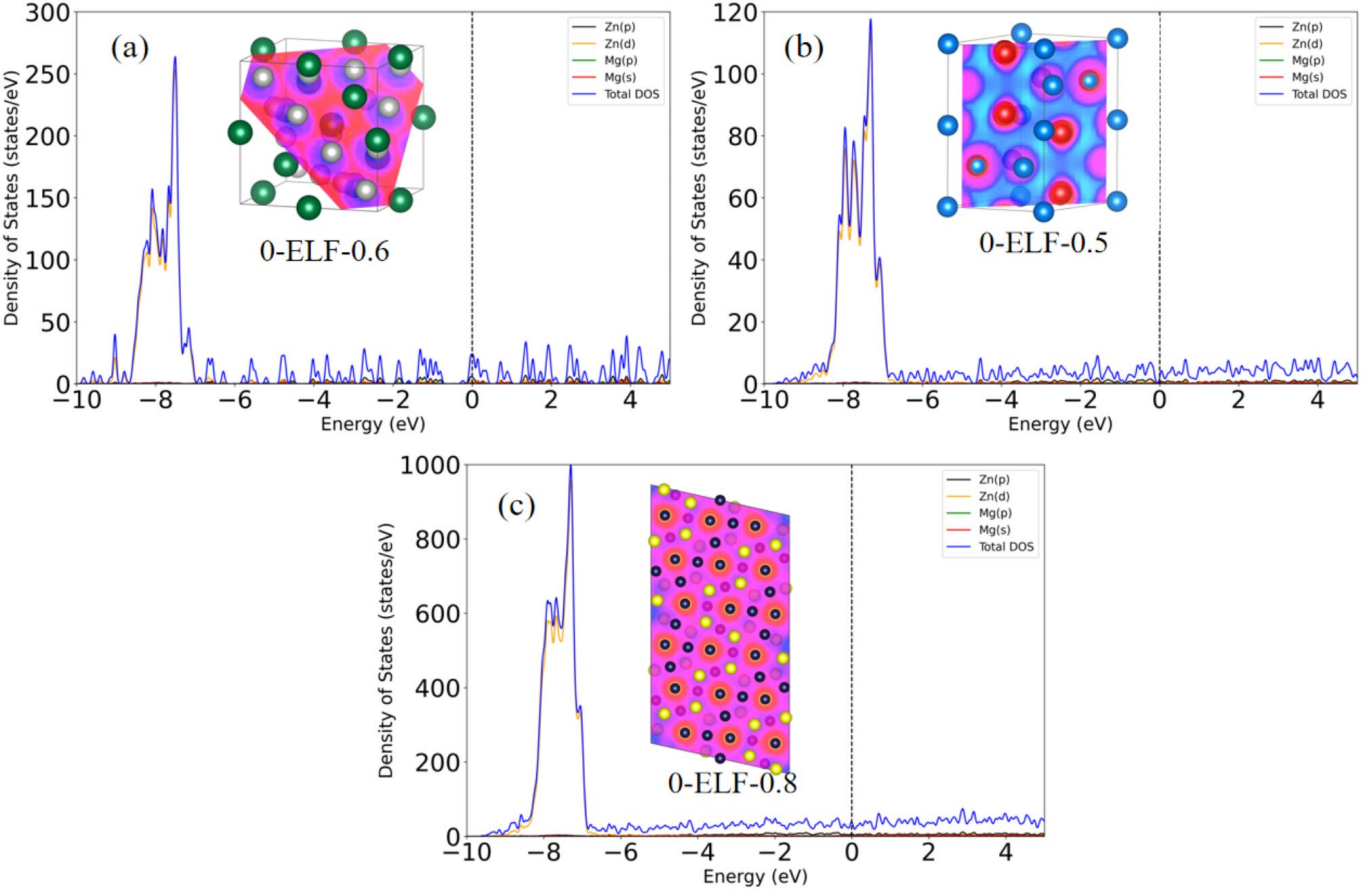
Electronic density of states (DOS) and electron localization function (ELF) of C-MgZn_2_ (**a**), H-MgZn_2_ (**b**), Mg_4_Zn_7_ (**c**).

From the density of states (DOS) diagrams, the DOS charts of the three phases show the distribution of electronic states near the Fermi level. For the C-MgZn_2_ phase (Fig. 2a): Near the Fermi level, the DOS shows a significant peak, indicating a large number of electronic states within this energy range. In addition, the ELF diagram demonstrates the degree of localization of electrons in the lattice structure, with a high pairing probability (ELF = 0.6) for the electron-like gas type, which is associated with covalent or metallic bonding in the crystal. The high DOS near the Fermi energy level as well as the relatively high bonding electrons in the C-MgZn_2_ phase suggest that the electronic structure favors the formation of stable chemical bonds, which enhances the stability of the system.

For the H-MgZn_2_ phase (Fig. 2b): close to the Fermi energy level, the peak of the density of states (DOS) decreases slightly, but remains significant. The electron localization function (ELF) plot shows the highest pairing probability (ELF = 0.5) for the electron-like gas type. This indicates the formation of strong ionic or metallic bonds between Mg-Zn in the H-MgZn_2_ phase^35^. Despite a slight decrease in the density of states, the high bonding electrons make the H-MgZn_2_ phase more stable^33^.

For the Mg_4_Zn_7_ phase (Fig. 2c): close to the Fermi energy level, the peak of the density of states (DOS) is similar to that of Fig. 2(a), and the stability of the Mg_4_Zn_7_ phase is similar to that of C-MgZn_2_. The electron localization function (ELF = 0.8) plot shows a relatively high degree of complete localization of electrons in the lattice structure, which suggests a more homogeneous distribution of electrons in the Mg_4_Zn_7_ phase, with weaker interactions between the electrons and a weaker ability to form bonds. As a result, the Mg_4_Zn_7_ phase is less stable. Although the difference in formation energies between these three phases does not exceed 0.3 KJ/mol, the difference in electronic structure leads to the difference in their stability.

**Fig. 3:**
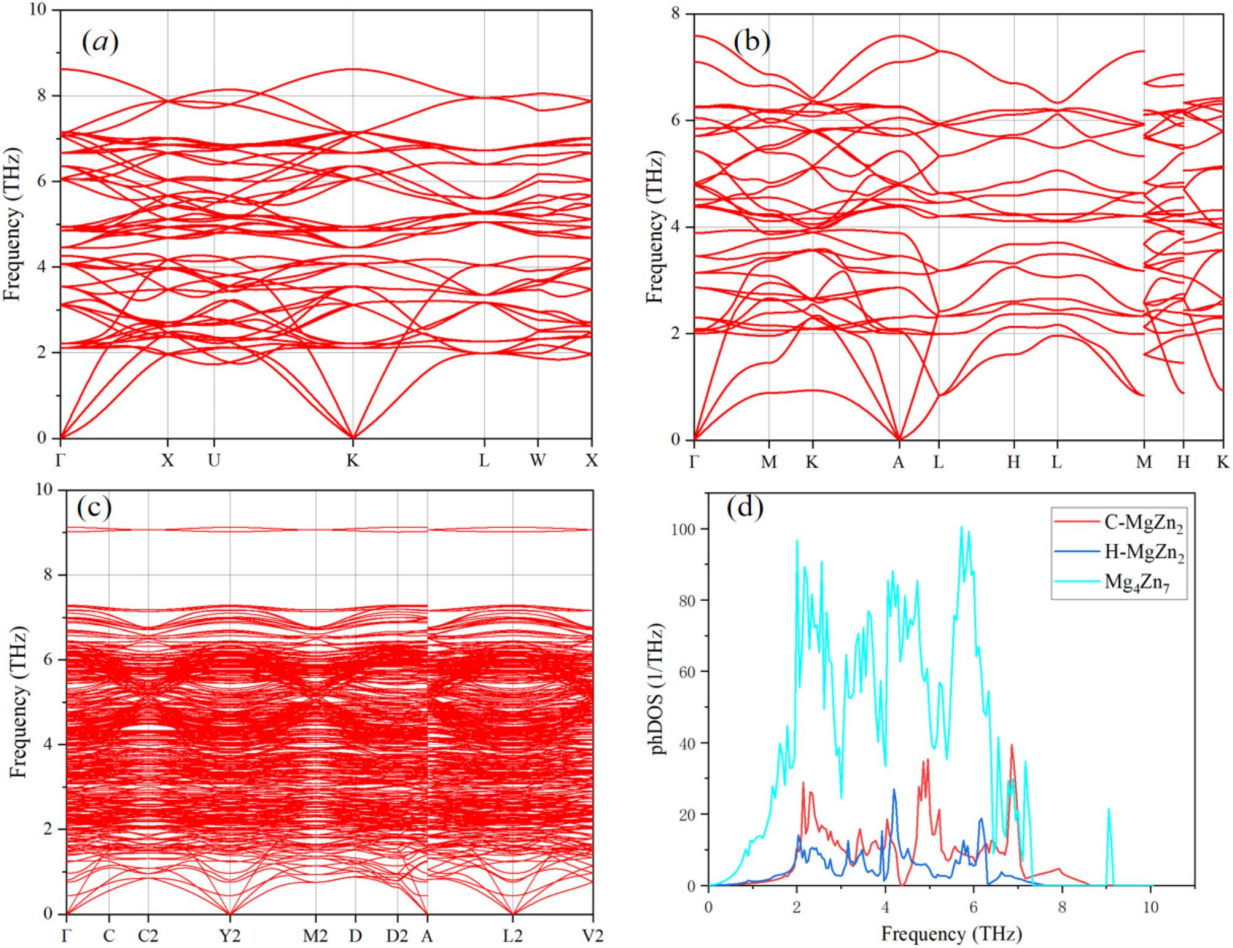
Phonon dispersion relations for C-MgZn_2_ (**a**), H-MgZn_2_ (**b**), and Mg_4_Zn_7_ (**c**) and their phonon density of states.

Figure 3 shows the phonon dispersion relations and phonon density of states for three different magnesium-zinc compounds (C-MgZn_2_, H-MgZn_2_, and Mg_4_Zn_7_). Figure 3(a) indicates that the phonon frequency distribution for each wave vector point ranges from 0 to approximately 10 THz, with a relatively dense dispersion relation. There are evident band crossings at some critical points, indicating that the energy levels of different phonon modes are close at these points, which may influence the thermal conductivity of the materials. In Fig. 3(b), the phonon dispersion range of H-MgZn_2_ is narrower, with frequencies concentrated from 0 to about 7 THz and significant band gaps, suggesting lower phonon scattering. Figure 3(c) shows that the phonon dispersion range of Mg_4_Zn_7_ is similar to that of C-MgZn_2_ but with denser dispersion curves, especially in the low-frequency range (0 to about 3 THz), indicating the presence of numerous low-frequency phonon modes.

Figure 3(d) displays multiple peaks in the phonon density of states curves for C-MgZn_2_ in the low-frequency range (0 to about 3 THz) and the high-frequency range (6 to approximately 10 THz). The density curve for H-MgZn_2_ has significant peaks in the low-frequency range (0 to about 2 THz) and the mid-to-high-frequency range (4 to approximately 7 THz). The density curve for Mg_4_Zn_7_ is the most complex, with multiple peaks in the low-frequency range (0 to about 3 THz), mid-frequency range (3 to 5 THz), and high-frequency range (6 to approximately 10 THz), indicating a large number of phonon modes across these frequency ranges.

In summary, C-MgZn_2_ and Mg_4_Zn_7_ have a greater number of phonon modes in the low-frequency and high-frequency ranges, while H-MgZn_2_ has significant phonon modes in the low-frequency and mid-frequency ranges. These differences may originate from their distinct crystal structures and are crucial for understanding the thermal conductivity and phonon scattering behavior of these materials. Contrary to the conventional view, our DFT-Debye analysis demonstrates that the bonding nature (ELF) and phonon mode distribution jointly determine phase dominance .

### Elastic anisotropy of the$\:{\:{\upbeta\:}}_{1}^{{\prime\:}}$-phase

**Table 2:** Elastic anisotropy factors of C-MgZn_2_, H-MgZn_2_, Mg_4_Zn_7_ phases.

	A	A_B_	A_G_	A^U^	A_1_	A_2_	A_3_	Reference
C-MgZn_2_	0.713	0	0.005	0.050	0.712	0.712	0.712	This work
H-MgZn_2_	2.617	0.022	0.125	1.479	1.017	1.017	0.991	This work
	-	0.005	0.040	-	0.513	0.513	1.0	Ref^36^.
	1.861	-	-	0.810	-	-	-	Ref^37^.
Mg_4_Zn_7_	0.816	0	0.011	0.109	0.750	0.955	0.832	This work
	0.659	-	-	0.150	-	-	-	Ref^37^.

As shown in Table 2, the elastic anisotropy of the C-MgZn_2_ phase is mild, but significant differences still exist at the microscopic scale. This anisotropy may affect its crystal structure and microstructure during phase transformation, such as the formation and distribution of rod-like precipitates ($\:{{\upbeta\:}}_{1}^{{\prime\:}}$ phases)^38^.

The elastic anisotropy factor A^U^ of the H-MgZn_2_ phase is 1.479, which is much greater than 0, indicating that the H-MgZn_2_ phase exhibits strong anisotropy overall.The anisotropy (A_3_) of H-MgZn_2_ in the [0001] direction is significantly lower than the other directions, leading to more susceptible elastic strain relaxation along the c-axis direction. In the early stages of aging, the dominance of rod-like precipitates may be related to their elastic behavior, may stabilize rod-like precipitates during early aging by minimizing interfacial strain energy along the [0001]α-Mg direction.According to the classical nucleation theory, the anisotropic strain energy difference (ΔU∝E^− 1^) will contribute to the extension of the precipitated phase along the low-modulus direction (c-axis) and the formation of a plate $\:{{\upbeta\:}}_{2}^{{\prime\:}}$-phase, which is consistent with the results of the over-aging experiments in the literature^39^ where H-MgZn_2_ plate phases prevailed.The elastic anisotropy factor A^U^ of the Mg_4_Zn_7_ phase is 0.109,indicating that it is also anisotropic overall. Although the degree of anisotropy of the Mg_4_Zn_7_ phase is not as strong as that of the H-MgZn_2_ phase, it is still significant.

**Fig. 4:**
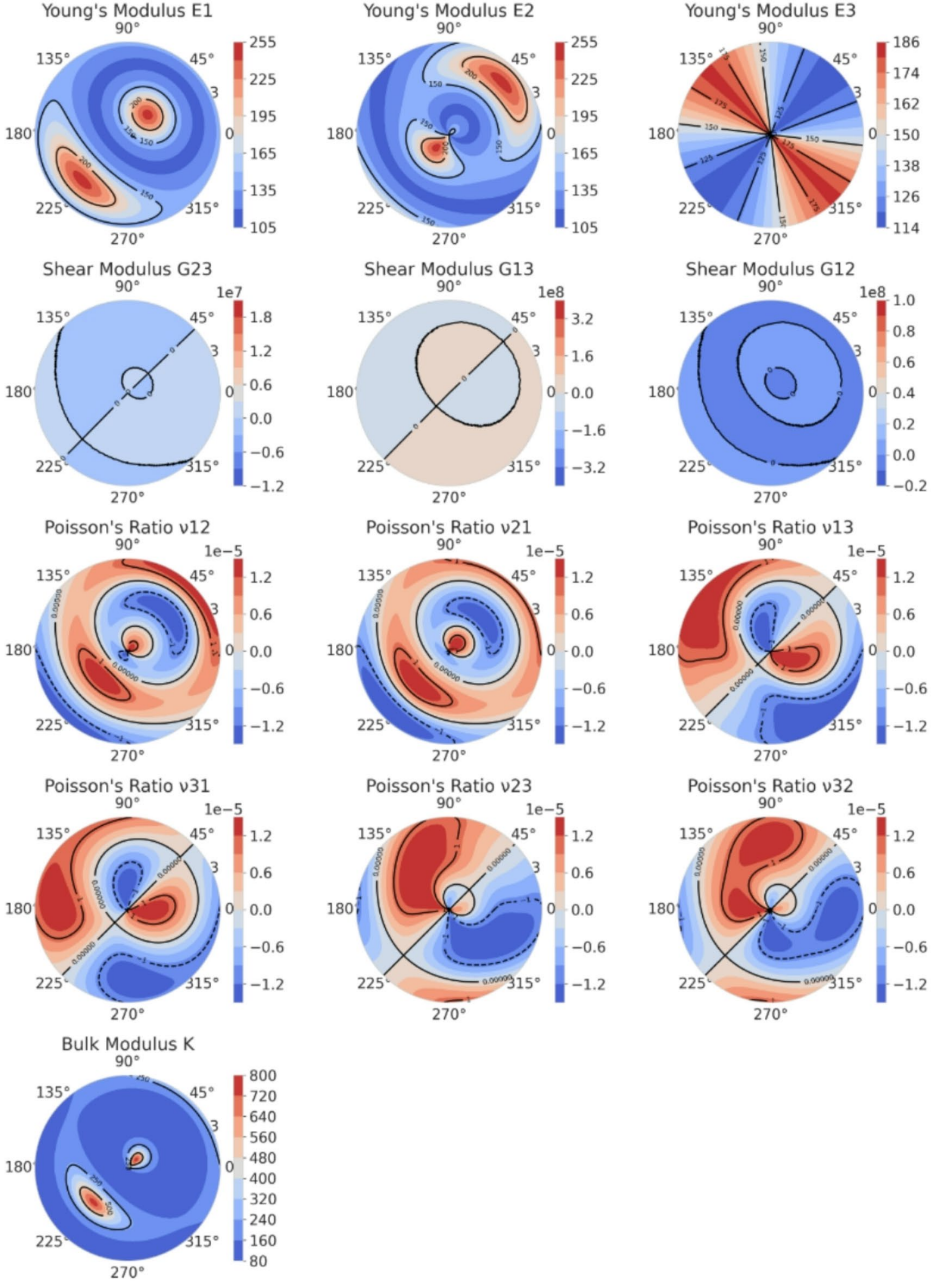
Polar contour plots of Young’s modulus (E), shear modulus (G), Poisson’s ratio (υ), and bulk modulus(K)of C-MgZn_2_.

**Fig. 5:**
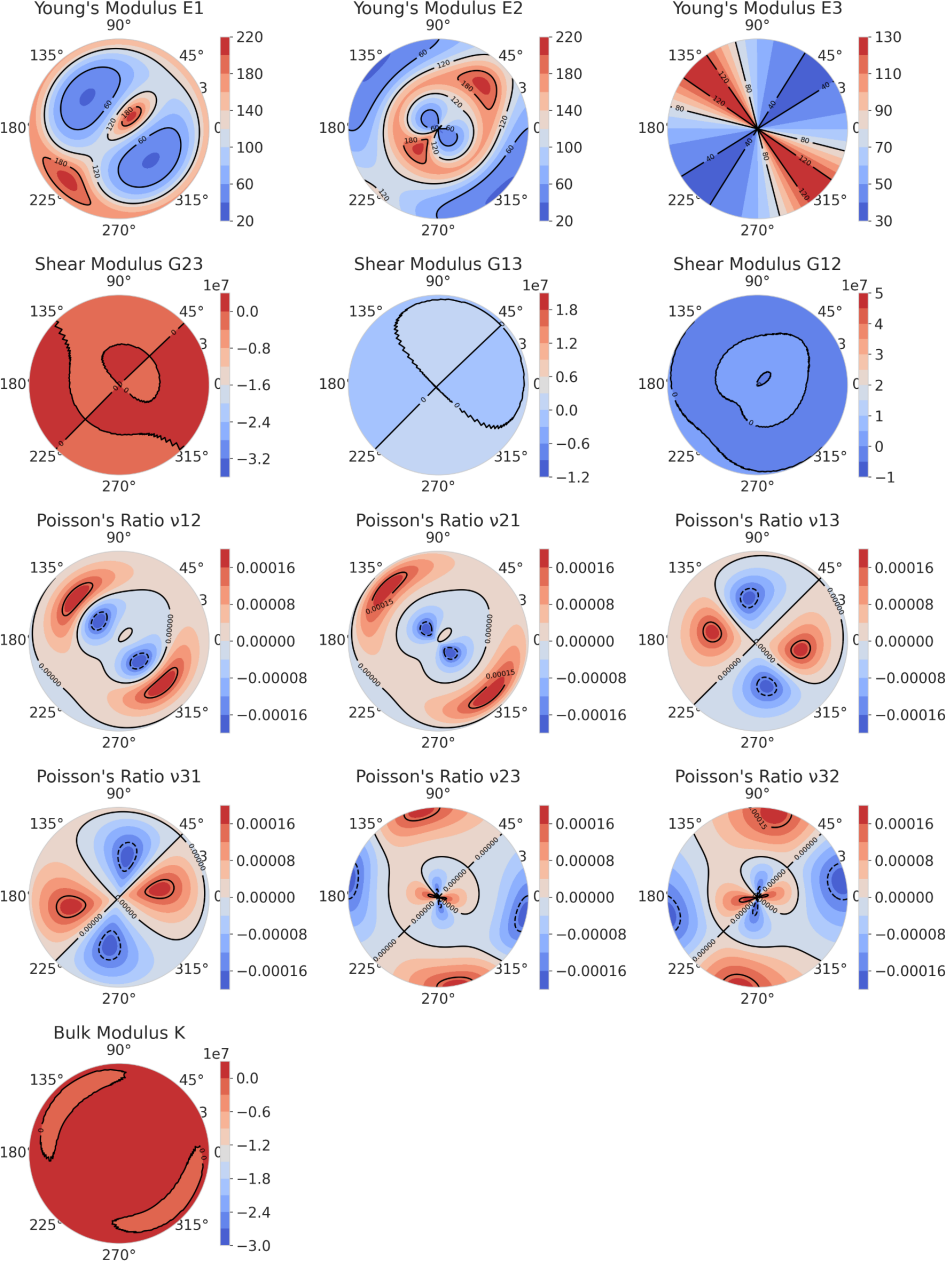
Polar contour plots of Young’s modulus (E), shear modulus (G), Poisson’s ratio (υ), and bulk modulus(K)of H-MgZn_2_.

**Fig. 6:**
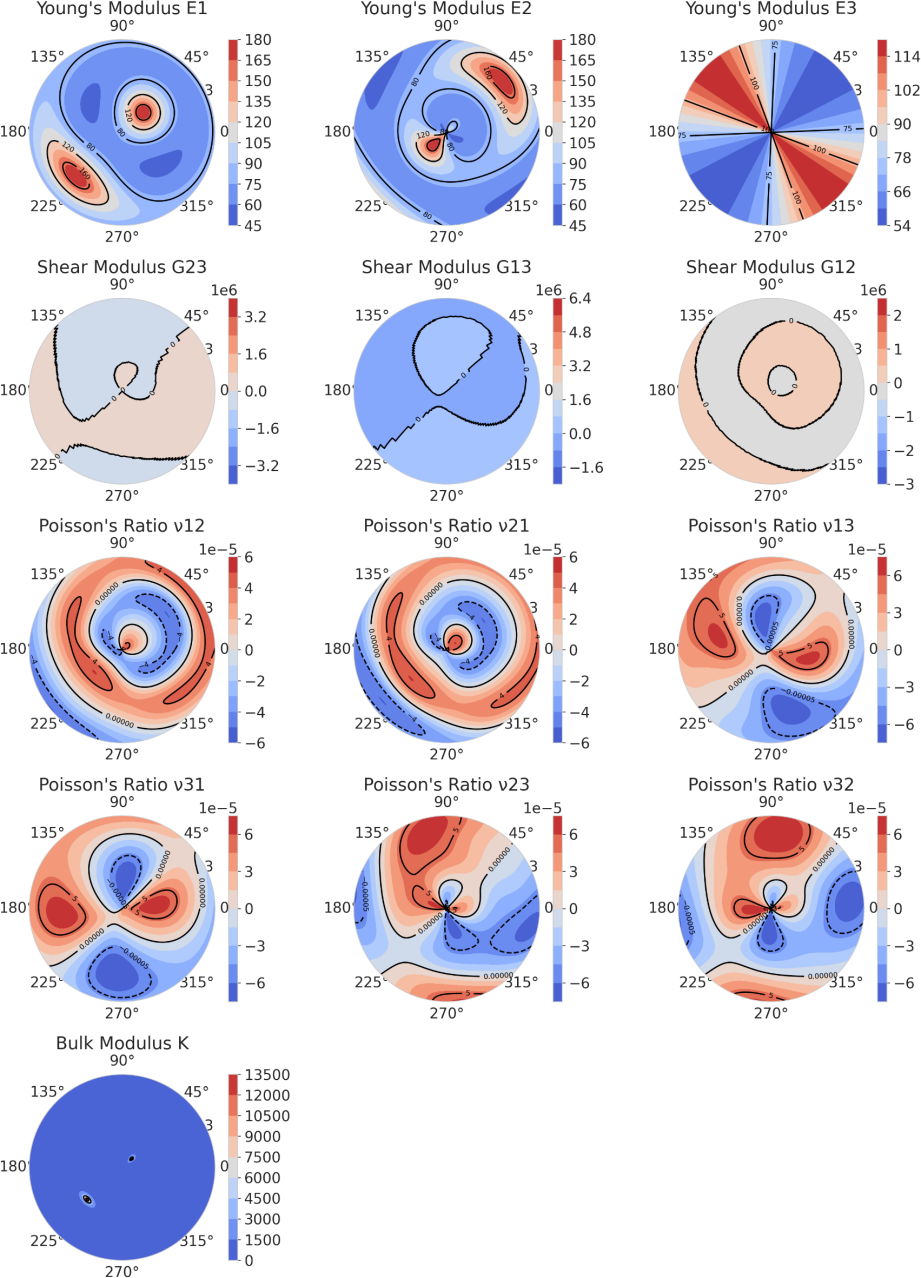
Polar contour plots of Young’s modulus (E), shear modulus (G), Poisson’s ratio (υ), and bulk modulus(K)of Mg_4_Zn_7_.

To more clearly analyze the elastic anisotropy of the three phases, the anisotropy of Young’s modulus (E), shear modulus (G), Poisson’s ratio (υ), and bulk modulus (K) for the C-MgZn_2_, H-MgZn_2_, and Mg_4_Zn_7_ phases is represented through Figs. 4 and 5, and 6, respectively.

From Fig. 4, it can be observed that the distribution of Young’s modulus (E) and shear modulus (G) for the C-MgZn_2_ phase shows a significant anisotropy. In the contour plot of Young’s modulus (E), the density and color variation of the contour lines in different directions indicates the changes in elastic modulus of the material in these directions. The plot shows that Young’s modulus is higher in some directions (such as 0° and 90°) and lower in others (such as 45° and 135°). This distribution pattern suggests that the material has a stronger resistance to external forces in these specific directions.

The contour plot of shear modulus (G) also demonstrates a clear anisotropy. In the contour plots of G23 and G13, the shear modulus is higher in certain specific directions and lower in others. This indicates that the material’s shear deformation capability varies in different directions. Among them, the distribution of G23 is relatively uniform, while in the distribution plots of G13 and G12, a higher shear modulus is visible near 0° and 90°, and a lower shear modulus near 45° and 135°. The contour plot of Poisson’s ratio (υ) shows how the ratio of lateral strain to axial strain changes in different directions for the C-MgZn_2_ phase. It is evident from Fig. 4 that there are significant differences in Poisson’s ratio in different directions. In the contour plots of υ13, υ31, υ23, and υ32, the pattern of changes is similar, indicating that the material’s deformation characteristics exhibit marked anisotropy in different directions across various types of Poisson’s ratio (the relationship between the longitudinal and transverse).

The contour plot of the bulk modulus (K) illustrates the material’s resistance to volumetric deformation in different directions. It is clear from the figure that the bulk modulus values differ significantly in different directions. In Fig. 4, the bulk modulus is higher in certain directions (such as 0° and 90°) and lower in other directions (such as 45° and 135°). This variation indicates that the material has a stronger resistance to external forces in these specific directions and weaker resistance in others.

Figure 5 shows the polar coordinate contour plots of Young’s modulus (E), shear modulus (G), Poisson’s ratio (υ), and bulk modulus (K) for the H-MgZn_2_ phase. It can be observed from Fig. 5 that the Young’s modulus of H-MgZn_2_ displays a notable anisotropy in different directions. The Young’s modulus in the three directions E1, E2, and E3 exhibits different distribution patterns, with E1 and E2 showing higher modulus values around 90°, and E3 showing relatively uniform values throughout the 360° range. This indicates that the phase has better elastic stability and consistency in the E3 direction, while it shows greater elastic variability in the E1 and E2 directions.

The shear modulus in the three directions G13, G23, and G12 also shows different distribution patterns from Fig. 5. The shear modulus in the G13 direction is relatively uniform across the 360° range, while the modulus in the G23 and G12 directions has higher values within specific angular ranges. This suggests that the phase has better shear stability in the G13 direction and exhibits more significant shear deformation in the G23 and G12 directions.

Figure 5 also exhibits Poisson’s ratio of H-MgZn_2_ in multiple directions, where the ratios in the v12, v21, v13, v23, v31, and v32 directions show different distribution patterns. Poisson’s ratio demonstrates a significant anisotropy in different directions, especially with the ratios in the v12 and v21 directions having higher values within certain angular ranges, indicating that the phase is prone to lateral contraction in these directions.

The bulk modulus (K), which reflects the material’s ability to resist volumetric strain, also shows a marked anisotropy in different directions according to Fig. 5. The bulk modulus is relatively uniform throughout the 360° range, suggesting that the phase has better volumetric stability in all directions.

Figure 6 shows the polar coordinate contour maps of the Young’s modulus (E), shear modulus (G), Poisson’s ratio (υ), and bulk modulus (K) of the Mg_4_Zn_7_ phase in different directions. The figure illustrates the Young’s modulus (E1, E2, E3) of the Mg_4_Zn_7_ phase in three different directions. From Fig. 6, it can be seen that there are significant differences in Young’s modulus in different directions. In the E1 direction: the Young’s modulus reaches its highest value of about 180 GPa at 135° and 315°, and its lowest value of about 45 GPa at 45° and 225°. In the E2 direction: the Young’s modulus reaches its highest value of about 180 GPa at 45° and 225°, and its lowest value of about 45 GPa at 135° and 315°. This indicates that the Young’s moduli in the E1 and E2 directions serve as complements to each other in symmetric angular distributions. In the E3 direction: the Young’s modulus is distributed relatively uniformly around the entire circumference, maintaining a value of about 114 GPa. This suggests that the Young’s modulus in the E3 direction is insensitive to changes in angle.

Figure 6 also shows the shear modulus (G23, G13, G12) of the Mg_4_Zn_7_ phase in three different shear directions. In the G23 direction: the shear modulus reaches its maximum value of about 3.2 GPa near 0° and 180°, and approaches 0 near 90° and 270°. In the G13 direction: the shear modulus reaches its maximum value of about 6.4 GPa near 90° and 270°, and approaches 0 near 0° and 180°. In the G12 direction: the shear modulus is distributed relatively uniformly around the entire circumference, maintaining a value of about 2 GPa. The Poisson’s ratio exhibits varying distributions in different directions. For instance, in the v12 direction, the Poisson’s ratio is close to 6 near 0° and 180°, and close to -6 near 90° and 270°. This indicates that forces applied in these directions result in corresponding maximum lateral contraction and expansion. Other Poisson’s ratio distributions show similar trends but with different directions and values. The bulk modulus is uniformly distributed around the entire circumference, maintaining a value of about 13,500 GPa. This indicates that the phase exhibits effective volumetric changes in response to forces applied in all directions.

The stark contrast in elastic anisotropy between C-MgZn_2_ and H-MgZn_2_ fundamentally governs their precipitation morphologies through strain energy minimization principles. For C-MgZn_2_, its near-isotropic elasticity (Young modulus in all directions, Figure.4) allows homogeneous nucleation of rod-like $\:{{\upbeta\:}}_{1}^{{\prime\:}}$ precipitates along the low-energy [0001]_α−Mg_ direction, as isotropic stiffness suppresses preferential growth directions. In contrast, H-MgZn_2_ exhibits extreme anisotropy with E1 (basal plane) vs. E3 (c-axis) (Figure.5). According to Eshelby’s inclusion theory^40^, the lower stiffness alongc-axis (E3/E1 = 0.63) reduces the strain energy penalty for plate-like $\:{{\upbeta\:}}_{1}^{{\prime\:}}$ precipitates extending along (0001) planes, consistent with TEM observations of {0001}_α−Mg_ habit planes in over-aged alloys^17^.

Additionally, the shear anisotropy factors (A_1_ = 1.017, A_2_ = 1.017, A_3_ = 0.991 for H-MgZn_2_) indicate that shear deformation preferentially occurs on (0001) planes (G12 vs. G13, Figure.5). This facilitates dislocation glide along these planes during aging, providing nucleation sites for plate-like precipitates. These mechanistic insights reconcile the long-debated correlation between $\:{{\upbeta\:}}_{1}^{{\prime\:}}$ phase anisotropy and precipitation morphology in Mg-Zn systems^16^.

### Thermodynamic properties of the $\:{{\upbeta\:}}_{1}^{{\prime\:}}$-phase

**Table 3:** Longitudinal speed of sound (v_l_), transverse speed of sound (v_t_), average speed of sound (v_m_), Débay temperature (${\theta _d}$), Débay velocity(${\omega _D}$), and Grüneisen parameter (γ) for C-MgZn_2_, H-MgZn_2_, Mg_4_Zn_7_ phase.

	v_l_ (km/s)	v_t_ (km/s)	v_m_ (km/s)	θ_d_	ω_D_(km/s)	γ
C-MgZn_2_	5.333	3.128	3.468	366.014	2.794	0.641
H-MgZn_2_	4.686	2.648	2.945	312.959	2.389	0.701
Mg_4_Zn_7_	4.450	2.409	2.688	287.938	2.198	0.768

The phonon velocity is a key physical quantity for assessing sound wave propagation in materials, reflecting density and elastic modulus. In Table 3, C-MgZn_2_ exhibits the highest sound velocity, indicating stronger interatomic forces and higher elastic modulus, which implies better rigidity and stability. H-MgZn_2_ has a slightly lower sound velocity, while Mg_4_Zn_7_ has the lowest, suggesting a more loosely packed crystal structure and weaker interatomic interactions, resulting in higher resistance to sound wave propagation. The average sound velocity (v_m_) in anisotropic materials can estimate thermal conductivity, reinforcing that C-MgZn_2_ has superior sound and thermal properties^41^.

The Debye temperature (θ_d_) and Debye velocity (ω_D_) are crucial parameters for lattice vibrations and phonon transport. C-MgZn_2_ has the highest Debye temperature (366.014 K) and Debye velocity (2.794 km/s), indicating stronger thermal conductivity and smaller atomic spacing. H-MgZn_2_ and Mg_4_Zn_7_ have lower values, particularly Mg_4_Zn_7_ with θ_d_ = 287.938 K and ω_D_ = 2.198 km/s, suggesting less efficient phonon transport.

The Grüneisen parameter describes temperature-dependent thermal properties. C-MgZn_2_ has the lowest Grüneisen parameter (0.641), indicating a smaller coefficient of thermal expansion and higher thermal conductivity. H-MgZn_2_ and Mg_4_Zn_7_ have higher parameters (0.701 and 0.768), suggesting larger thermal expansion and lower thermal conductivity.

**Fig. 7:**
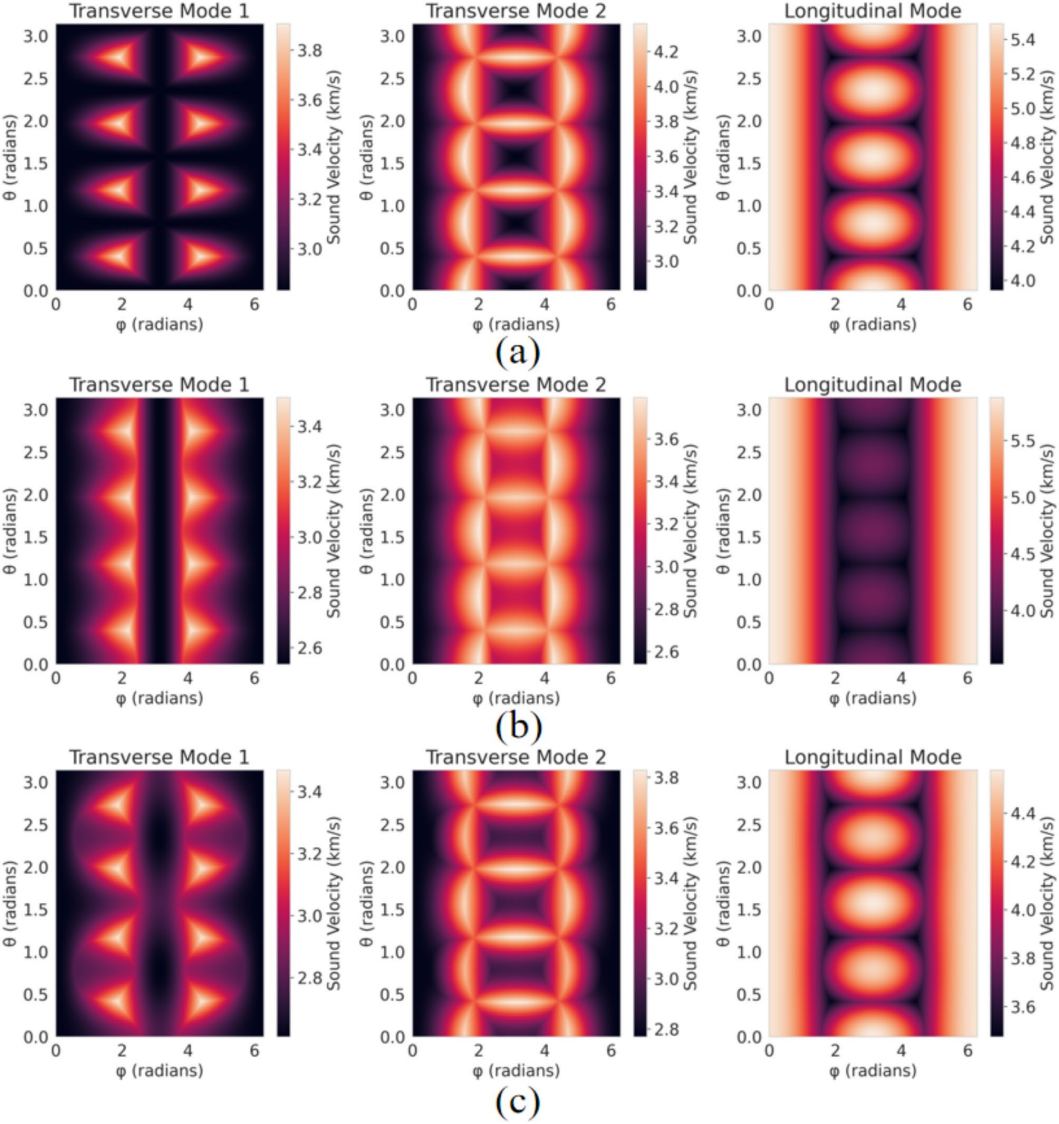
Grouped phonon velocity thermograms for C-MgZn_2_, H-MgZn_2_, Mg_4_Zn_7_.

Figure 7 shows the distribution of phonon velocities for C-MgZn_2_, H-MgZn_2_, and Mg_4_Zn_7_ materials at different angles θ and φ. Each chart is composed of three subplots, representing two transverse phonon modes (Transverse Mode 1 and Transverse Mode 2) and one longitudinal phonon mode (Longitudinal Mode). The color bar indicates the range of phonon velocities, varying from 3.0 km/s to 5.5 km/s.

In Fig. 7(a)-(c), Transverse Mode 1 shows distinct peaks at specific angles with phonon velocities ranging from 2.8 to 3.8 km/s. Transverse Mode 2 exhibits more uniform distributions, with velocities from 3.0 to 4.2 km/s, showing enhancements at certain angles. The Longitudinal Mode has relatively uniform distributions with velocities from 3.6 to 5.5 km/s, also featuring noticeable enhancements at specific angles.

**Fig. 8:**
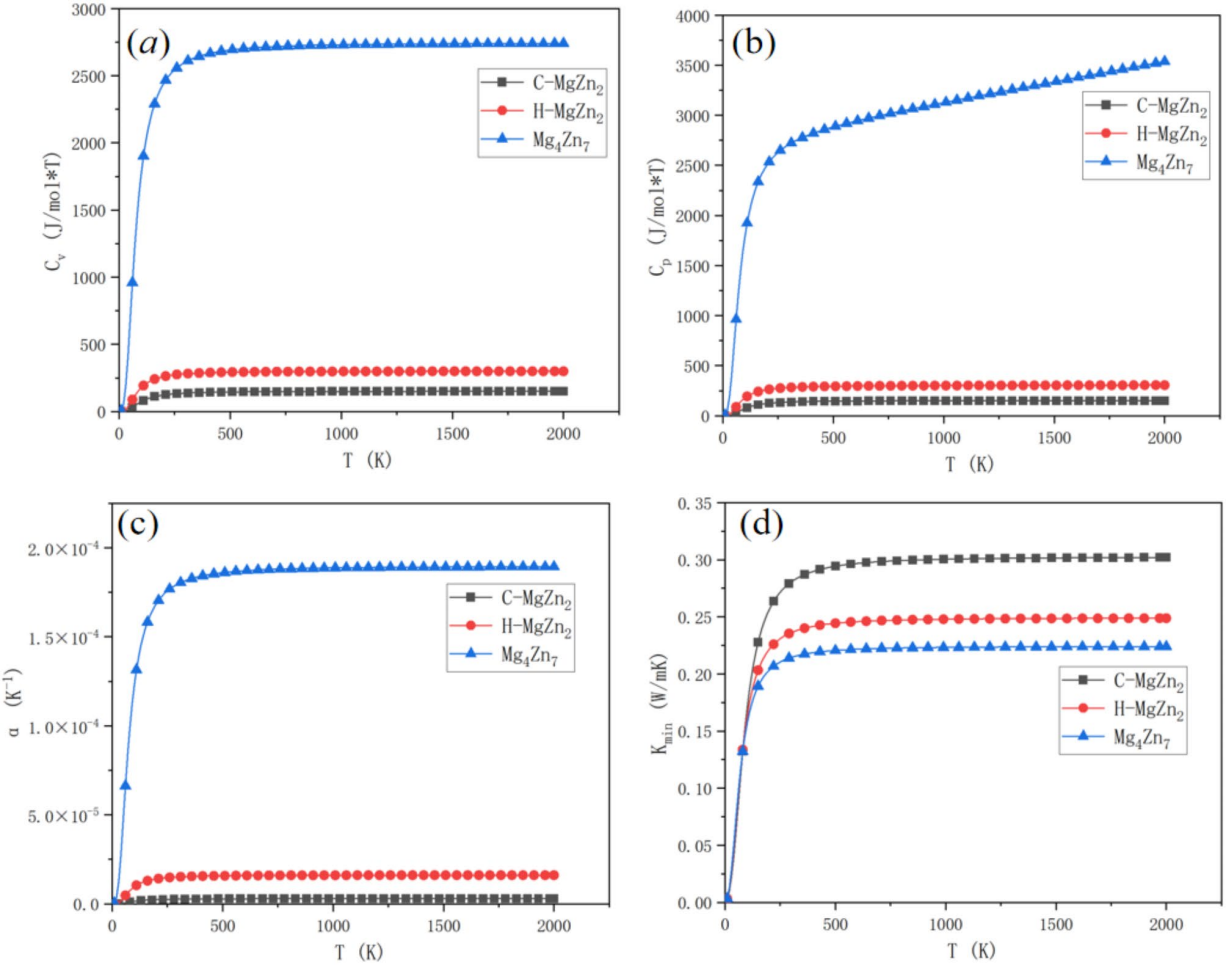
Isomeric heat capacity (**a**), isobaric heat capacity (**b**), thermal expansion (**c**) and thermal conductivity (**d**) of C-MgZn_2_, H-MgZn_2_, Mg_4_Zn_7_.

Figure 8(a) shows the temperature-dependent isochoric heat capacity (C_v_) for C-MgZn_2_, H-MgZn_2_, and Mg_4_Zn_7_. C-MgZn_2_’s C_v_ rises sharply from near 0 K, moderates, and plateaus around 288.15 K, consistent with the Debye model. H-MgZn_2_ follows a similar trend but remains lower, suggesting more complex lattice vibrations and less heat absorption. Mg_4_Zn_7_’s C_v_ is significantly higher, rising rapidly at low temperatures and stabilizing around 2700 J/mol·K, indicating more low-frequency lattice modes and greater thermal energy absorption.

Figure 8(b) presents the temperature-dependent isobaric heat capacity (C_p_) for the three phases. C-MgZn_2_’s C_p_ increases sharply from near 0 K, moderates, and plateaus around 288.15 K, aligning with the Debye model. Mg_4_Zn_7_’s C_p_ shows a gradual upward trend, reflecting the thermal excitation of lattice vibrations. At low temperatures, only low-frequency modes are excited, causing a rapid increase in C_p_. As temperature rises, more modes are excited, leading to a linear increase and eventual stabilization.

Figure 8(c) shows the thermal expansion rates of C-MgZn_2_, H-MgZn_2_, and Mg_4_Zn_7_. C-MgZn_2_ has a very low thermal expansion rate, approximately 8 × 10^− 6^ K^− 1^, indicating minimal volume change with temperature. H-MgZn_2_’s rate is slightly higher at around 3.5 × 10^− 5^ K^− 1^, showing moderate thermal expansion. Mg_4_Zn_7_ has the highest rate, sharply increasing from low temperatures and stabilizing at about 1.7 × 10^− 4^ K^− 1^, indicating significant volume expansion when heated.

Figure 8(d) shows the thermal conductivities of C-MgZn_2_, H-MgZn_2_, and Mg_4_Zn_7_ as a function of temperature. All three phases exhibit a sharp rise from near 0 K to about 2000 K, then level off. C-MgZn_2_ has the highest thermal conductivity, stabilizing at around 0.30 W/m·K, due to its highest average sound velocity (v_m_), Debye temperature (θ_d_), and Debye frequency (ω_D_), which reduce long-range phonon scattering. H-MgZn_2_ and Mg_4_Zn_7_ have lower thermal conductivities, stabilizing at 0.25 W/m·K and 0.20 W/m·K, respectively. The lower Grüneisen parameter (γ) in C-MgZn_2_ further reduces lattice-phonon scattering, enhancing thermal conductivity.

## Conclusion

While prior studies have predominantly focused on the structural characterization of individual $\:{{\upbeta\:}}_{1}^{{\prime\:}}$ phase components (e.g., C14 MgZn_2_, monoclinic Mg_4_Zn_7_ ), the novelty of this work lies in the first systematic comparison of three critical $\:{{\upbeta\:}}_{1}^{{\prime\:}}$ phase variants (Mg_4_Zn_7_, C-MgZn_2_, H-MgZn_2_) through integrated electronic-phonon-thermodynamic analysis, revealing their structure-dependent anisotropy and stability mechanisms. Specifically, we uncover that:

1. C-MgZn_2_ and H-MgZn_2_ exhibit stronger chemical bonding stability compared to Mg_4_Zn_7_, suggesting their higher phase stability in Mg-Zn alloys.Distinct vibrational patterns are observed: C-MgZn_2_ and Mg_4_Zn_7_ exhibit enhanced phonon modes at low and high frequencies, whereas H-MgZn_2_ is dominated by medium-frequency modes, reflecting fundamental differences in lattice dynamics.
2. The near-isotropic elasticity of C-MgZn_2_ (A^U^=0.05) versus the extreme anisotropy of H-MgZn_2_ (A^U^=1.479) provides a mechanistic basis for their distinct precipitation morphologies (rod-like vs. plate-like).
3. C-MgZn_2_ displays superior thermal stability (θ_d_ = 366 K, v_m_ = 3.468 m/s, γ = 0.641), indicating high thermal conductivity and low thermal vibration dissipation. In contrast, Mg4Zn7 has the highest thermal expansion coefficient (α = 3.0 × 10^− 5^K^−^¹), which may limit its high-temperature applications.

## Data Availability

The authors ensure that the data supporting the findings of the study are available to Zhiyong You (youzhiy1486@163.com) and can be provided if needed.

## References

[1] Li, P., Hou, D., Han, E. H., Chen, R. & Shan, Z. Solidification of Mg–Zn–Zr alloys: Grain growth restriction, dendrite coherency and grain size. *Acta Metall. Sin (Engl Lett)*. **33**, 1477–1486 (2020).

[2] Zhang, J. et al. Dynamic precipitation and enhanced mechanical properties of ZK60 magnesium alloy achieved by low temperature extrusion. *Mater. Sci. Engineering: A*. **829**, 142143 (2022).

[3] Jin, S. et al. First-principles study of Y, Ca microalloyed Mg-Zn alloy. *Mater. Today Commun.* **41**, 110936 (2024).

[4] Bao, L. et al. First-principles study on the interfacial bonding strength and segregation at Mg/MgZn2 matrix interface. *J. Magnesium Alloys*. 10.1016/j.jma.2022.12.010 (2023).

[5] Ma, H. T. et al. The role of Ag, Ca, Zr and al in strengthening effects of ZK series alloys by altering G.P. Zones stability. *Acta Mater.* **147**, 42–50 (2018).

[6] Nie, J. F., Wilson, N. C., Zhu, Y. M. & Xu, Z. Solute clusters and GP zones in binary Mg–RE alloys. *Acta Mater.* **106**, 260–271 (2016).

[7] Gao, X. & Nie, J. F. Characterization of strengthening precipitate phases in a Mg–Zn alloy. *Scripta Mater.* **56**, 645–648 (2007).

[8] Gao, X. & Nie, J. F. Structure and thermal stability of primary intermetallic particles in an Mg–Zn casting alloy. *Scripta Mater.* **57**, 655–658 (2007).

[9] Singh, A. & Tsai, A. P. Structural characteristics of Β1′ precipitates in Mg–Zn-based alloys. *Scripta Mater.* **57**, 941–944 (2007).

[10] Rosalie, J. M., Somekawa, H., Singh, A. & Mukai, T. Orientation relationships between icosahedral clusters in hexagonal MgZn2 and monoclinic Mg4Zn7 phases in Mg-Zn(-Y) alloys. *Phil. Mag.* **91**, 2634–2644 (2011).

[11] Xie, H. et al. Self-Assembly of two unit cells into a nanodomain structure containing Five-Fold symmetry. *J. Phys. Chem. Lett.* **9**, 4373–4378 (2018). 30028626 10.1021/acs.jpclett.8b01526

[12] Bendo, A. et al. Characterisation of structural similarities of precipitates in Mg–Zn and Al–Zn–Mg alloys systems. *Phil. Mag.* **99**, 2619–2635 (2019).

[13] Wang, D. et al. Crystal structure, energetics, and phase stability of strengthening precipitates in Mg alloys: A first-principles study. *Acta Mater.* **158**, 65–78 (2018).

[14] Liu, S., Esteban-Manzanares, G. & LLorca, J. First-principles analysis of precipitation in Mg-Zn alloys. *Phys. Rev. Mater.* **4**, 093609 (2020).

[15] Cheng, D., Wang, K. & Zhou, B. C. First-Principles investigation of the Early-Stage precipitations in Mg-Sn and Mg-Zn alloys. in Magnesium Technology 2022 (eds Maier, P., Barela, S., Miller, V. M. & Neelameggham, N. R.) 281–290 (Springer International Publishing, Cham, doi:10.1007/978-3-030-92533-8_47. (2022).

[16] Cheng, D., Wang, K. & Zhou, B. C. Crystal structure and stability of phases in Mg-Zn alloys: A comprehensive first-principles study. *Acta Mater.* **242**, 118443 (2023).

[17] Yang, Y. et al. Revisiting precipitation kinetics in Mg-Zn alloy – a multi-characterization and modeling study. *Acta Mater.* **260**, 119276 (2023).

[18] *Anisotropy in Single-Crystal Refractory Compounds*.

[19] Chung, D. H. & Buessem, W. R. The elastic anisotropy of crystals. *J. Appl. Phys.* **38**, 2010–2012 (1967).

[20] Ranganathan, S. I. & Ostoja-Starzewski, M. Universal elastic anisotropy index. *Phys. Rev. Lett.* **101**, 055504 (2008). 18764407 10.1103/PhysRevLett.101.055504

[21] Chen, S. B., Chen, Y., Yan, W. J. & Gao, T. H. First-principles investigation of elastic anisotropy and thermal transport property of transition metal monosilicides CrSi, TiSi, and ZrSi under pressure. *Mater. Today Commun.* **39**, 108958 (2024).

[22] Tao, X. et al. Calculation of the thermodynamic properties of B2 AlRE (RE = Sc, Y, La, Ce–Lu). *Phys. B*. **399**, 27–32 (2007).

[23] Cahill, D. & Pohl, R. Lattice vibrations and heat transport in crystals and glasses. *Annu. Rev. Phys. Chem.* **39**, 93–121 (2003).

[24] Kresse, G. & Furthmüller, J. Efficient iterative schemes for Ab initio total-energy calculations using a plane-wave basis set. *Phys. Rev. B*. **54**, 11169–11186 (1996). 10.1103/physrevb.54.11169 9984901

[25] Blöchl, P. E. Projector augmented-wave method. *Phys. Rev. B*. **50**, 17953–17979 (1994). 10.1103/physrevb.50.17953 9976227

[26] Perdew, J. P., Burke, K. & Ernzerhof, M. Generalized gradient approximation made simple. *Phys. Rev. Lett.* **77**, 3865–3868 (1996). 10062328 10.1103/PhysRevLett.77.3865

[27] Pfrommer, B. G., Côté, M., Louie, S. G. & Cohen, M. L. Relaxation of crystals with the Quasi-Newton method. *J. Comput. Phys.* **131**, 233–240 (1997).

[28] Togo, A., Chaput, L., Tadano, T. & Tanaka, I. Implementation strategies in phonopy and phono3py. *J. Phys. : Condens. Matter*. **35**, 353001 (2023). 10.1088/1361-648X/acd831 37220761

[29] Liu, Z. L. et al. Investigating elastic constants across diverse strain-matrix sets. *Comput. Mater. Sci.* **230**, 112521 (2023).

[30] Ekuma, C. E. & Liu, Z. L. ElasTool v3.0: Efficient computational and visualization toolkit for elastic and mechanical properties of materials. *Comput. Phys. Commun.* **300**, 109161 (2024).

[31] Momma, K. & Izumi, F. VESTA 3 for three-dimensional visualization of crystal, volumetric and morphology data. *J. Appl. Cryst.* **44**, 1272–1276 (2011).

[32] Mao, P., Yu, B., Liu, Z., Wang, F. & Ju, Y. Mechanical properties and electronic structures of MgCu2, Mg2Ca and MgZn2 Laves phases by first principles calculations. *Trans. Nonferrous Met. Soc. China*. **24**, 2920–2929 (2014).

[33] Song, Y. et al. First-Principles investigations on structural stability, elastic properties and electronic structure of Mg32(Al,Zn)49 phase and MgZn2 phase. *Crystals* **12**, 683 (2022).

[34] Jain, A. et al. Commentary: the materials project: A materials genome approach to accelerating materials innovation. *APL Mater.* **1**, 11002 (2013).

[35] Becke, A. D. & Edgecombe, K. E. A simple measure of electron localization in atomic and molecular systems. *J. Chem. Phys.* **92**, 5397–5403 (1990).

[36] Wu, M. M., Wen, L., Tang, B. Y., Peng, L. M. & Ding, W. J. First-principles study of elastic and electronic properties of MgZn2 and ScZn2 phases in Mg–Sc–Zn alloy. *J. Alloys Compd.* **506**, 412–417 (2010).

[37] Xie, Y. P., Wang, Z. Y. & Hou, Z. F. The phase stability and elastic properties of MgZn2 and Mg4Zn7 in Mg–Zn alloys. *Scripta Mater.* **68**, 495–498 (2013).

[38] Rosalie, J. M. & Pauw, B. R. Form-free size distributions from complementary Stereological TEM/SAXS on precipitates in a Mg–Zn alloy. *Acta Mater.* **66**, 150–162 (2014).

[39] Li, X. D. et al. First-principles study of coherent interfaces of Laves-phase MgZn2 and stability of thin MgZn2 layers in Mg-Zn alloys. *J. Alloys Compd.* **696**, 109–117 (2017).

[40] Lurie, S., Volkov-Bogorodsky, D., Leontiev, A. & Aifantis, E. Eshelby’s inclusion problem in the gradient theory of elasticity: applications to composite materials. *Int. J. Eng. Sci.* **49**, 1517–1525 (2011).

[41] Wang, K. et al. First-principles investigations on the electronic structures, polycrystalline elastic properties, ideal strengths and elastic anisotropy of U3Si2. *Eur. Phys. J. Plus*. **136**, 409 (2021).

